# The GSK461364 PLK1 inhibitor exhibits strong antitumoral activity in preclinical neuroblastoma models

**DOI:** 10.18632/oncotarget.14268

**Published:** 2016-12-27

**Authors:** Kristian W Pajtler, Natalie Sadowski, Sandra Ackermann, Kristina Althoff, Kerstin Schönbeck, Katharina Batzke, Simonäfers Sch, Andrea Odersky, Lukas Heukamp, Kathy Astrahantseff, Annette Künkele, Hedwig E Deubzer, Alexander Schramm, Annikaüssel Spr, Theresa Thor, Sven Lindner, Angelika Eggert, Matthias Fischer, Johannes H Schulte

**Affiliations:** ^1^ Department of Physiology, Medical School, Institute for Medical Sciences, Chonbuk National University, Jeonju, Republic of Korea; ^2^ Department of Pediatric Oncology, Hematology and Immunology, University Hospital, Heidelberg, Germany; ^3^ German Cancer Consortium (DKTK Core Center Heidelberg), Germany; ^4^ Department of Pediatric Oncology and Hematology, University Children`s Hospital Essen, Essen, Germany; ^5^ Department of Pediatric Oncology and Hematology, University Children's Hospital, and Center for Molecular Medicine Cologne (CMMC), Cologne, Germany; ^6^ Department of Pediatric Oncology/Hematology, Charité-Universitätsmedizin Berlin, Germany; ^7^ NEO New Oncology, Cologne, Germany; ^8^ Institute for Hematopathology, Hamburg, Germany; ^9^ Berlin Institute of Health (BIH), Germany; ^10^ German Cancer Consortium (DKTK Berlin), Germany; ^11^ German Cancer Consortium (DKTK Essen), Germany; ^12^ Translational Neuro-Oncology, West German Cancer Center, University Hospital Essen, University Duisburg-Essen, Essen, Germany; ^13^ Medical Faculty, University of Cologne, Cologne, Germany; ^14^ German Cancer Research Center (DKFZ), Heidelberg, Germany

**Keywords:** polo-like kinase 1, pediatric solid tumors, targeted therapy, *MYCN*

## Abstract

Polo-like kinase 1 (PLK1) is a serine/threonine kinase that promotes G2/M-phase transition, is expressed in elevated levels in high-risk neuroblastomas and correlates with unfavorable patient outcome. Recently, we and others have presented PLK1 as a potential drug target for neuroblastoma, and reported that the BI2536 PLK1 inhibitor showed antitumoral actvity in preclinical neuroblastoma models. Here we analyzed the effects of GSK461364, a competitive inhibitor for ATP binding to PLK1, on typical tumorigenic properties of preclinical *in vitro* and *in vivo* neuroblastoma models. GSK461364 treatment of neuroblastoma cell lines reduced cell viability and proliferative capacity, caused cell cycle arrest and massively induced apoptosis. These phenotypic consequences were induced by treatment in the low-dose nanomolar range, and were independent of *MYCN* copy number status. GSK461364 treatment strongly delayed established xenograft tumor growth in nude mice, and significantly increased survival time in the treatment group. These preclinical findings indicate PLK1 inhibitors may be effective for patients with high-risk or relapsed neuroblastomas with upregulated PLK1 and might be considered for entry into early phase clinical trials in pediatric patients.

## INTRODUCTION

Neuroblastoma is the most common extracranial solid tumor in childhood [1]. It is characterized by a broad biological heterogeneity based on molecular genetic variations in *MYCN* oncogene copy number, chromosomal ploidy changes or partial losses and gains, alterations in neurotrophin receptor expression that correlate to different degrees with clinical outcome [2] and with recurrent mutations in a few genes [3]. Clinical course ranges from complete spontaneous regression or differentiation of low-stage or stage 4s neuroblastomas, even without therapeutic intervention, to widespread metastatic disease that is refractory to aggressive multimodal therapies. Although treatment of solid tumors in childhood has significantly improved over the past decades, overall survival in high-risk neuroblastoma patients remains less than 40% despite intensive therapy regimens [1]. While 20% of neuroblastomas harbor *MYCN* amplifications, directly classifying them as high-risk, around 50% of high-risk tumors lack *MYCN* amplification and display molecular diversity within the high-risk tumor group [1, 4]. Since 40 to 50% of patients initially diagnosed with neuroblastoma must be assigned to the high-risk group [5, 6], treatment of this disease remains challenging for pediatric oncologists and novel therapeutic options are still urgently needed.

Loss of cell cycle regulatory control is a major hallmark of many cancers, including neuroblastoma [7, 8]. The serine/threonine kinase, polo-like kinase 1 (PLK1), is an essential regulator promoting entry into the mitotic phase from the G2/M transition point in the cell cycle of nontransformed cells and after a DNA damage checkpoint arrest [9–11]. There is increasing evidence that elevated PLK1 activity might serve as a tumor-promoting force by stimulating mitotic transcriptional programs to evade the DNA damage checkpoint [12, 13]. PLK1 expression is higher in cancer cells than in nontransformed cells, and promotes G1/S transition and DNA replication in addition to the G2/M phase transition [14–16]. PLK1 dysregulation is initiated early in carcinogenesis, and promotes cellular processes necessary for oncogenesis and enhances pro-oncogenic signaling networks, including TP53 and RB1 [17–20].

A wide variety of cancers including entities predominantly occurring in children overexpress PLK1 [21–29]. PLK1 was identified as one of the most important survival kinases for rhabdomyosarcoma in a genome-wide siRNA library screen [21]. Inhibiting PLK1 in xenografts or cell lines deriving from osteosarcoma and medulloblastoma, another embryonal tumor of childhood, suppressed proliferation and induced apoptosis [22–24, 29]. Abbou and colleagues recently demonstrated preclinical efficacy of PLK1 inhibition in a wide panel of pediatric malignancies independent of tumor histology [30]. PLK1 upregulation in primary neuroblastomas strongly correlates with high-risk disease [6]. We and others have previously demonstrated that PLK1 is a potential therapeutic target in neuroblastoma, and that inhibition with the small molecule, BI2536, effectively decreased growth in cell and mouse models [6, 31]. Normal, but not cancer, cells have previously been shown to survive PLK1 depletion [32]. A small molecule screen to identify kinase inhibitors that suppress neuroblastoma tumor initiating cell (TIC) proliferation identified PLK1 as a promising target whereas only exceedingly high inhibitor concentrations were cytotoxic for neural stem cells in this screen [33]. These results indicate that targeting this aberrant mitotic kinase signaling pathway in precision therapies that combine targeted drugs and standard chemotherapy could benefit patients with high-risk neuroblastoma.

The three PLK1 inhibitors currently furthest in clinical development are the dihydropteridinone derivatives, BI2536 and BI6727 (volasertib), and the imidazotriazine, GSK461364 [34–37]. All three are competitive inhibitors of ATP-kinase binding. GSK461364 treatment produced fewer side effects related to toxicity than BI2536. Side effects in patients treated with GSK461364 included vein thromboses in patients not co-treated with low molecular weight heparin and mild myelotoxicity [36, 38]. GSK461364 treatment at half-maximal inhibitory concentrations (IC50) below 100 nM inhibited proliferation in multiple tumor cell lines [35, 39, 40]. Here we evaluated the ability of GSK461364 to inhibit neuroblastoma cell viability and proliferation and to induce death in cell lines with different *MYCN* copy number backgrounds, and to suppress xenograft tumor growth in nude mice.

## RESULTS

### PLK1 pathway inhibition by GSK461364 reduces viability and clonogenicity of neuroblastoma cells

**Figure 1:**
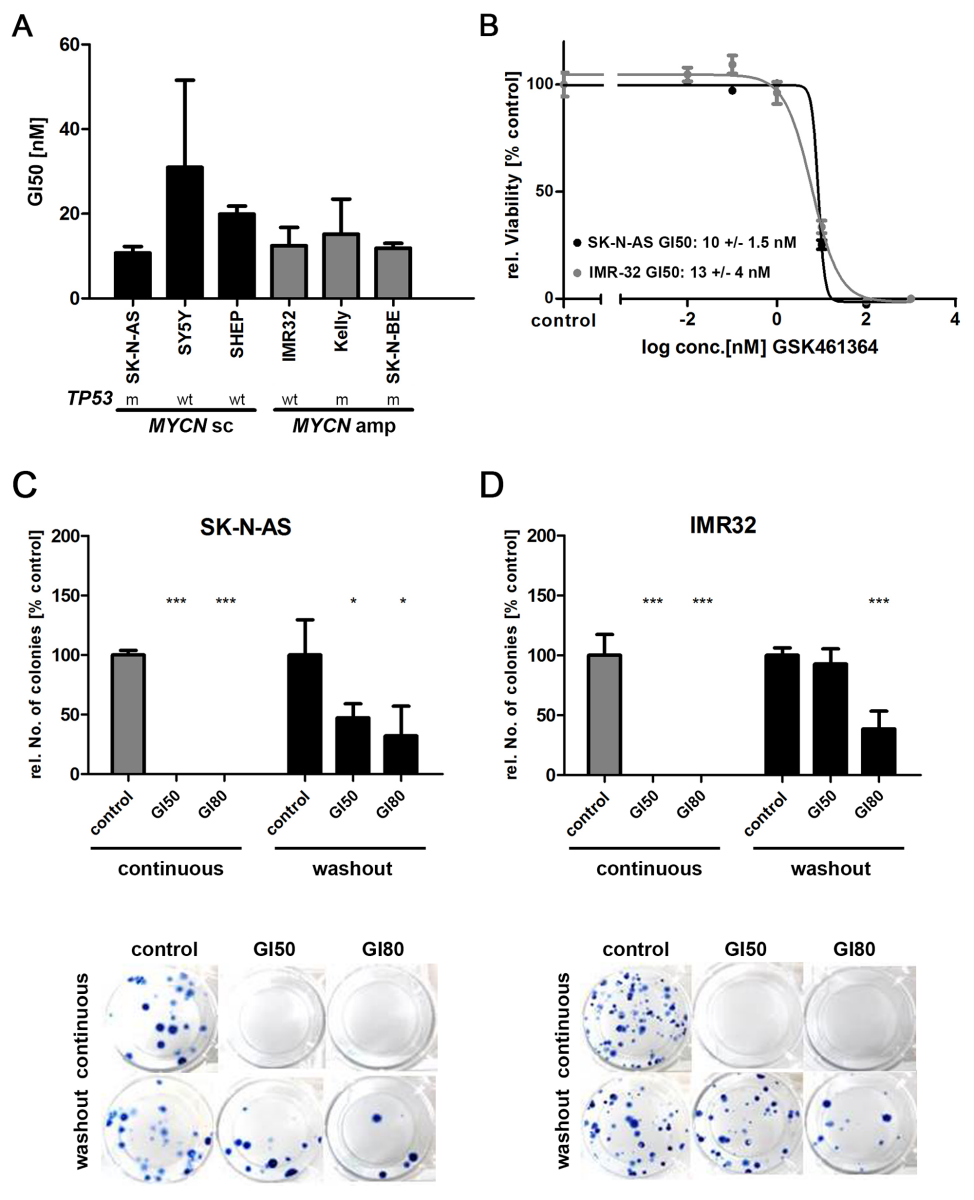
GSK461364-mediated PLK1 inhibition reduces cell viability and clonogenicity in neuroblastoma cell lines **A**. Neuroblastoma cell lines were treated with 0.01-1000nM GSK461364 or control medium containing equivalent DMSO carrier concentrations for 72 hours, then cell viability was measured using the MTT assay. Bars represent GI50 values calculated for each cell line. *MYCN* status (sc = single copy; amp = amplified) and *TP53* status (wt = wild-type; m = mutated) of cell lines is indicated. **B**. Representative dose-response curves of neuroblastoma cell lines SK-N-AS and IMR32, with single copy and amplified *MYCN* status, respectively. Cells were treated with indicated concentrations of GSK461364 for 72 hours and changes in cell viability relative to solvent-treated cultures was measured by the MTT assay. **C**. SK-N-AS or **D**. IMR32 cell lines were either continuously treated over 10 days with GSK461364 at GI50 or GI80 or GSK461364 was relieved from the cells after incubation for 24 hours by repeated replacement of the cell culture medium (wash out). Colony forming ability was assessed for both experimental settings after 10 days using a clonogenic cell survival assay. Solvent-treated cultures (0.1% DMSO) were used as control. ***p<0.001, *p<0.05

Neuroblastoma cell lines have previously been shown to express high PLK1 protein levels, similar to expression in primary tumors [6]. To survey a broad range of aggressive neuroblastoma subtype backgrounds, we selected neuroblastoma cell lines either lacking (SK-N-AS, SH-SY5Y, SH-EP) or harboring (Kelly, IMR32, SK-N-BE) *MYCN* amplifications for *in vitro* analyses. We assessed cellular viability using MTT assays. GSK461364 treatment significantly reduced viability of neuroblastoma cells in culture (Figure 1A and 1B). Fifty percent inhibition of growth (GI50) was observed at inhibitor concentrations below 20 nM in our cell line panel. Viability was comparably suppressed in all cell lines, regardless of whether they harbored *MYCN* amplifications or *TP53* mutations. To insure that GSK461364 was inhibiting PLK1 activity in our experiments, we assessed expression and phosphorylation of target proteins downstream of PLK1 in treated and untreated SK-N-AS and IMR-32 cells comprising MST1, MST2, pMST1/2, WEE1 and pWEE1 (Supplementary Figure 1). We next assessed clonal expansion in the SK-N-AS (*MYCN* single copy) and IMR32 (*MYCN*-amplified) cell lines treated with GSK461364 in colony forming assays. Continuous exposure of the SK-N-AS and IMR32 cell lines to GI50 or GI80 GSK461364 concentrations for 10 days fully inhibited colony formation (Figure 1C and 1D). Pulsed GSK461364 treatment for only 24 hours with GI80 still reduced colony formation by more than 50% after 10 days in culture (Figure 1C and 1D). Our experiments show that GSK461364-mediated PLK1 inhibition significantly reduced neuroblastoma cell viability and colony-forming ability *in vitro*, even when applied in short-term treatment pulses. These effects were independent of the molecular background imparted by *MYCN* and *TP53* status.

### PLK1 inhibition reduces proliferation and induces cell cycle arrest and apoptosis in neuroblastoma cells

**Figure 2:**
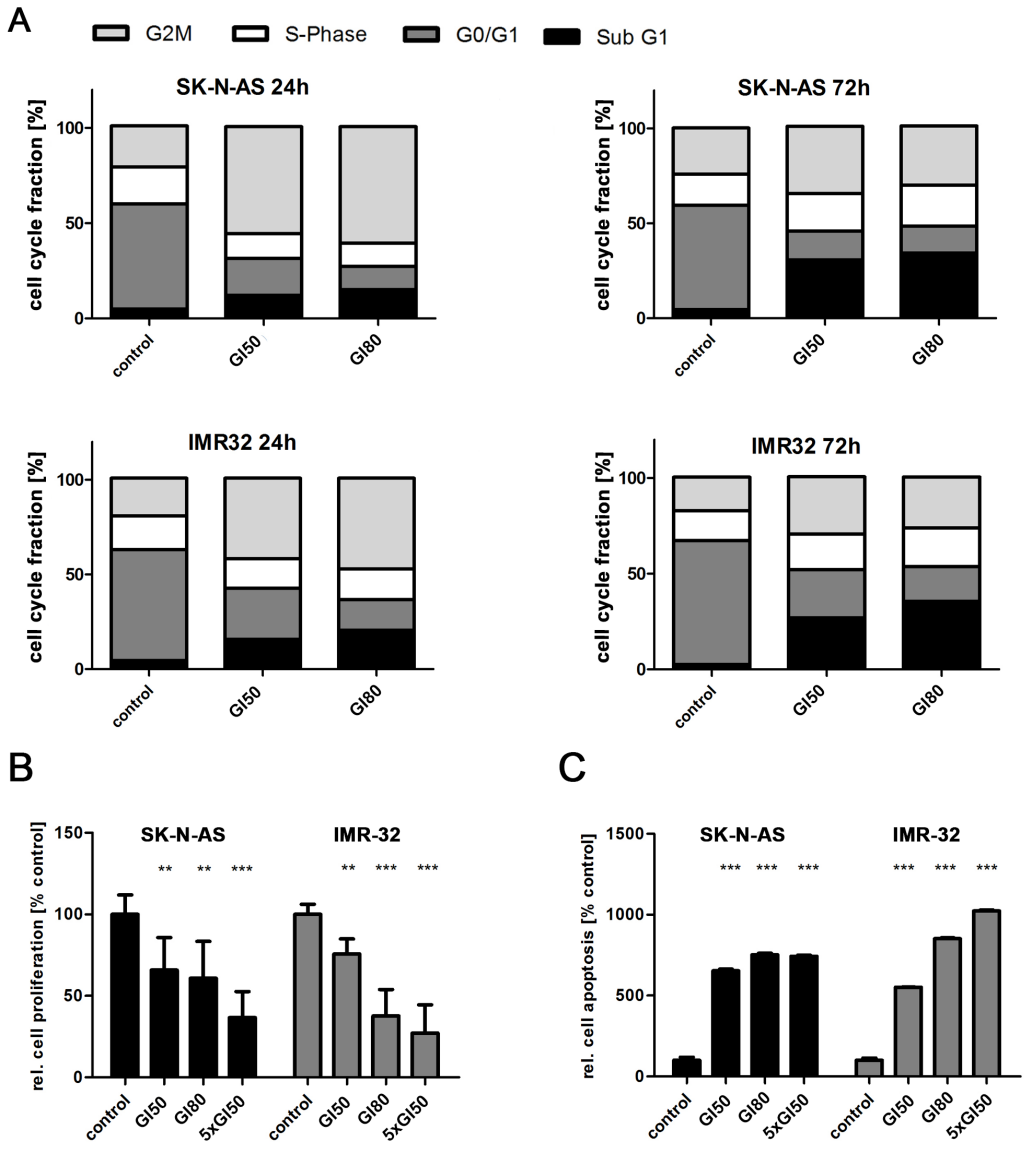
Inhibiting PLK1 with GSK461364 suppresses proliferation and induces both cell cycle arrest and apoptosis in neuroblastoma cells **A**. Fractions of SK-N-AS and IMR32 neuroblastoma cells in each point of the cell cycle measured by flow cytometry after 24 and 72 hours of treatment with GSK461364 at GI50 or GI80 or with solvent as control. **B**. BrdU ELISA performed after GSK461364 treatment of SK-N-AS and IMR32 neuroblastoma cells at GI50, GI80 or 5-fold GI50 for 24 hours. Bar graph shows mean proliferation (±SD) in relation to control (0.1% DMSO). **p<0.01, ***p<0.001 **C**. Degree of apoptosis in SK-N-AS and IMR32 neuroblastoma cells in relation to control after 24 hours of treatment with GI50, GI80 or 5-fold GI50 of GSK461364 detected by cell death ELISA (mean ±SD). ***p<0.001.

We next explored how GSK461364 inhibits neuroblastoma cell viability. We assessed proliferation and apoptosis in the SK-N-AS and IMR32 cell lines after short- and long-pulsed GSK461364 treatment, and flow cytometrically monitored cell cycle phase distribution in treated versus control cell cultures. Flow cytometric analyses were conducted after 24 and 72 hours of GSK461364 treatment. A larger proportion of cells treated with GI50 or GI80 concentrations of GSK461364 were in G2/M compared to untreated cultures (Figure 2A). These results are consistent with a previous report that PLK1 activity is necessary for mitotic entry from G2/M arrest induced by DNA damage [41], and indicate that GSK461364-mediated PLK inhibition prevents neuroblastoma cell cycle progression. The effects of GSK461364 on G2/M arrest and apoptosis in neuroblastoma cells did not appear to be influenced by the cellular *MYCN* background (single copy vs. amplified). BrdU incorporation 24 hours after beginning GSK461364 treatment at GI50 concentrations was significantly reduced in SK-N-AS and IMR32 cells compared with untreated cultures (Figure 2B). Proliferation, as measured by BrdU incorporation, was also further reduced by treating cells with the GI80 or 5-fold GI50 GSK461364 concentrations. GSK461364 treatment also increased the proportion of sub-G1 cells, which includes apoptotic cells (Figure 2A). Treatment at GI80 resulted in slightly more sub-G1 cells than treatment at GI50, and the effect was most pronounced after 72 hours of treatment. We confirmed that GI50 GSK461364 concentrations significantly induced apoptosis in SK-N-AS and IMR32 cells using the cell death detection ELISA™ (Figure 2C), and apoptotic cell fractions rose further when GSK461364 concentrations were increased to GI80 or 5-fold GI50. These data demonstrate that GSK461364 inhibits proliferation and induces both cell cycle arrest at the G2/M restriction point and apoptosis in neuroblastoma cells, regardless of *MYCN* status.

### Cell cycle regulators are differentially expressed following PLK1 inhibition by GSK461364

**Figure 3:**
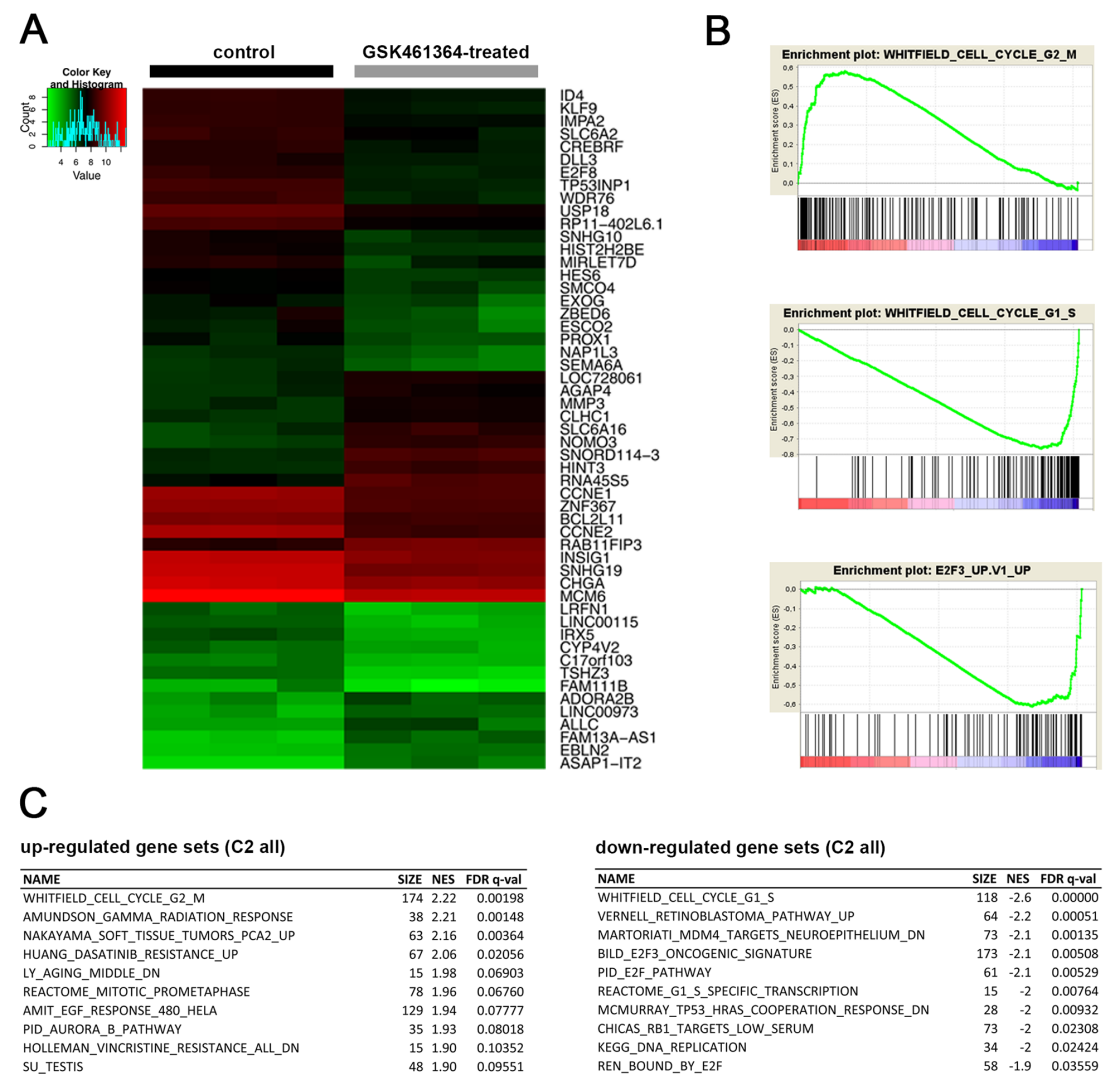
Cell cycle regulators are differentially expressed following GSK461364-mediated PLK1 inhibition **A**. Heatmap resulting from unsupervised hierarchical clustering of samples treated with GSK461364 for 24 hours (n = 3) and controls (n = 3). Only the top 50 differentially expressed genes are displayed. **B**. GSEA plots showing enrichment for genes involved in G2/M-phase (top) and downregulation of genes associated with G1/S-phase (middle) as well as with the E2F3 transcription factor (bottom). **C**. The top 10 up- and downregulated gene sets.

We analyzed gene expression profiles from treated and untreated SK-N-AS cells to investigate molecular mechanisms behind the GSK461364 influence on the neuroblastic cell cycle. Unsupervised hierarchical clustering clearly separated untreated from GSK461364-treated cells (Figure 3A). Gene set enrichment analyses identified increased expression of genes associated with G2/M and a decline in expression of genes essential for S-phase transition (Figure 3B and 3C). Three gene sets associated with the E2F transcription factor family, E2F3 and E2F1 were among the most downregulated in GSK461364-treated cells (Figure 3B, 3C and Supplementary Table 1 and 2). We also validated E2F1 and E2F3 downregulation in GSK461364-treated SK-N-AS and IMR-32 cells using western blotting (Supplementary Figure 1). We also applied GSEA analysis for cancer-related signatures to the expression data from GSK461364-treated and untreated SK-N-AS cells. Genes known to be upregulated by RB1 tumor suppressor loss (gene set RB_DN.V1_UP) were downregulated in GSK461364-treated cells compared to untreated controls (Supplementary Table 2). Since RB1 regulates the G1/S transition via E2F transcription factors [42, 43], these results are in line with our observed drop in gene expression associated with S-phase transition. We conclude that GSK461364 treatment not only induces expression patterns associated with a G2/M arrest, but might also inhibit G1/S transition via the RB1-E2F axis in neuroblastoma cells.

### GSK461364-mediated PLK1 inhibition has antitumoral activity against human neuroblastoma xenografts in mice

**Figure 4:**
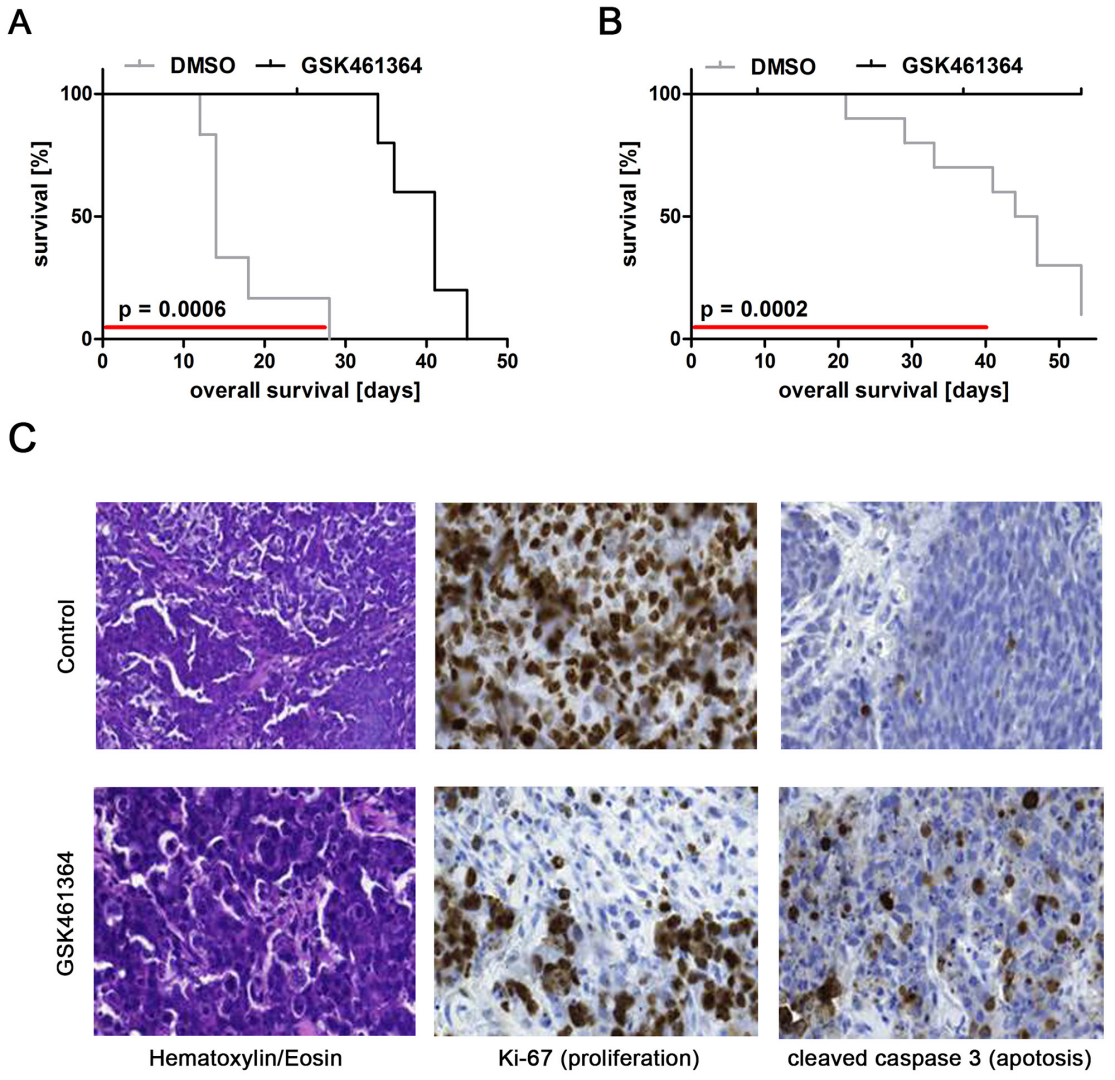
GSK461364-mediated PLK1 inhibition exerts antitumoral activity against human neuroblastoma xenografts in mice Kaplan-Meier analysis of GSK461364-treated and control mouse cohorts in animals injected with **A**. SK-N-AS or **B**. IMR-32 cells. Red lines indicate duration of treatment. **C**. Micrographs show immunostainings for Ki67 to identify proliferating cells and cleaved caspase 3 to identify apoptotic cells in addition to hematoxylin & eosin staining (HE) for SK-N-AS xenograft tumors 3 days after start of treatment.

To investigate GSK461364 efficacy *in vivo*, we treated a xenograft model for high-risk neuroblastoma grown subcutaneously in nude mice. The SK-N-AS cell line lacks *MYCN* amplification and is derived from a bone marrow metastasis from a patient with stage 4 disease [44]. SK-N-AS cells were injected subcutaneously into the flanks of immunocompromised mice, and xenograft tumor development was monitored until they reached 200 μl in volume. GSK461364 (50 mg / kg body weight) or vehicle alone (control group) was intraperitoneally administered once daily to 6 mice per group. GSK461364 treatment significantly reduced tumor volume compared to controls (Supplementary Figure 2A). Kaplan-Meier analysis showed that treatment delayed tumor growth to 2,500 mm^3^ by 22 days (time when the first tumor in the control group reached 2,500 mm^3^ compared to when the first tumor in the treatment group reached 2,500 mm^3^, Figure 4A), thus, significantly increasing survival in the treatment cohort. One of 6 mice in the treatment group had to be euthanized due to cachexia. Mice harboring subcutaneous xenograft tumors formed from *MYCN*-amplified IMR32 cells were also treated using the same GSK461364 or control injection regimen. IMR32 xenograft tumors developed considerably slower than SK-N-AS xenograft tumors. GSK461364 treatment also significantly reduced IMR32 xenograft tumor growth compared to the control group (Figure 4B, Supplementary Figure 2B). Immunohistological examination of xenograft tumors showed that GSK461364 reduced the number of proliferating cells and increased the number of apoptotic cells in tumors (Figure 4C). Our data clearly demonstrate that GSK461364 treatment *in vivo* suppresses growth of xenografts derived from neuroblastoma cells with high-risk characteristics regardless of *MYCN* status.

## DISCUSSION

Treating patients with high-risk neuroblastoma remains challenging, since further escalation of existing therapies is limited by toxicity [45]. Encorporating targeted drugs into existing approaches should lower toxicity while better inhibiting growth characteristics of high-risk neuroblastomas. Here we present preclinical data for the efficacy of the PLK1 inhibitor, GSK461364, against models for high-risk neuroblastoma. GSK461364 inhibited neuroblastoma cell viability *in vitro* via suppressing proliferation and preventing progression through cell cycle blocks at G2 and possibly G1. GSK461364 induced apoptosis *in vitro* and suppressed xenograft tumor growth in mice. GSK461364 inhibited neuroblastoma cell growth regardless of *MYCN* copy number status and presence or absence of *TP53* mutations.

High-level PLK1 expression has previously been reported in a variety of pediatric cancers [6, 22, 46]. Aggressive neuroblastoma subtypes overexpressed both PLK1 transcripts and protein [6]. PLK1 overexpression is known to override the G2/M and spindle checkpoints induced by DNA damage, thereby promoting chromosome instability and aneuploidy and fostering cancer progression [47]. We here show that inhibiting PLK1 with GSK461364 effectively reverted these effects, causing G2/M arrest accompanied by reduced proliferation and apoptosis in neuroblastoma cells. The expression profiling we conducted with and without GSK461364 treatment also indicated that PLK1 inhibition represses the E2F1 and E2F3 transcriptional activators, which promote G1/S transition [48]. Interestingly, miR-34a, which acts as a tumor suppressor in neuroblastoma, has also been shown to downregulate E2F factors and is suggested to directly suppress PLK1 translation [49–51]. Effects of miR-34a are comparable to those caused by GSK461364 treatment here. E2F family deregulation especially occurs in recurrent neuroblastomas and tumor-derived cell lines, presumably caused by upregulation of factors, such as CDK4, that inhibit the RB1 tumor suppressor [7, 52, 53]. Our analyses of signaling pathways associated with oncogenesis, also showed that GSK461364 treatment restored gene expression associated with unperturbed RB1 function. We conclude that PLK1 inhibition blocks cell cycle progression in neuroblastic cells by blocking PLK1 canonical regulatory function at the G2/M transition as well as cancer cell-specific blockage of G1/S transition occurring presumably via de-repression of RB1-associated gene expression.

Some genetic backgrounds have been proposed to sensitize cancer cells to PLK1 inhibition. Gorlick and colleagues treated a panel of predominantly *MYCN*-amplified neuroblastoma cell lines grown as xenograft tumors in CB17SC scid^−/−^ mice with the PLK1 inhibitor, volasertib [54]. They observed a ≥2-fold increase in the time to event in treated mice, and speculated that the enhanced *MYCN*-driven cell cycle progression might help sensitize neuroblastoma cells to agents that block mitotic progression. In contrast, in our study also GSK461364 treatment of neuroblastoma cells lacking *MYCN* amplifications and expressing low levels of *MYCN* significantly inhibited proliferation, induced apoptosis and caused cell cycle arrest in *in vitro*, and significantly reduced *in vivo* tumor growth of neuroblastoma cells independent of *MYCN* copy number status. Degenhardt and colleagues treated a panel of cell lines derived from adult cancers with GSK461364A, and concluded that cancer cell sensitivity to PLK1 inhibition is mediated by loss of TP53 functionality [55]. *TP53* is rarely mutated in neuroblastomas. However, a TP53 variant (p53ΔC) lacking the nuclear localization signal and part of the oligomerization domain [56], and a truncated TP53 isoform (p53β) [57] being also present in SK-N-AS cells, were reported for neuroblastoma. The neuroblastoma cell lines tested here harbor both wildtype or mutated *TP53* (cf. Figure 1A), but were comparably sensitive to low nanomolar GSK461364 concentrations. We conclude that GSK461364 produces antineoplastic effects in neuroblastoma cells independent of *MYCN* background or TP53 functional status.

Targeted therapies can be circumvented by resistance mechanisms developed by cancer cells during tumor progression. Gilmartin and colleagues treated mice bearing colorectal (Colo205) xenograft tumors with several GSK461364A dosing schedules [35]. Xenograft tumor volume was reduced by all schedules during the treatment period, but resumed growth afterwards. Antimitotic drugs either enhance antiapoptotic protein expression to keep cell fractions alive during mitotic arrest or act cytotoxically depending on cellular context and inhibitor concentration [58, 59]. Cells in stasis might resume proliferation after treatment. Overexpression of the human multidrug resistance gene, *ABCB1*, was also recently reported to reduce GSK461364 activity in cancer cells [60]. Future in-depth preclinical studies addressing whether neuroblastic tumors regrow after PLK1-inhibiting treatment ceases and identifying the resistance mechanisms involved should assist schedule refinement for incorporating PLK1 inhibitors into current protocols for high-risk or relapsed disease. Ferrarotto and colleagues recently observed different sensitivities of cancer cell lines comparing treatments with dihydropteridinone derivatives, BI2536 or volasertib, with GSK461364 [34]. They suggested that higher sensitivity to GSK461364 might result from increased PLK1 selectivity of this compound compared to the two other inhibitors. It remains to be further elucidated if one class of PLK1 inhibitors might be substituted by another in case of resistance. To minimize resistance development, PLK1 inhibitors will most likely be combined with other synergizing targeted agents or conventional chemotherapeutics in treatment regimens. To date, synergism has especially been shown for PLK1-inhibitor based combination therapies with microtubule-interfering drugs (e.g. vincristine) [30, 61–63]. In contrast, the PLK1 inhibitor volasertib showed antagonistic effects when combined with etoposide in a cell line panel of pediatric malignancies [30].

Here we explored the feasibility of treating high-risk neuroblastoma patients with GSK461364 in preclinical models. GSK461364 suppressed proliferation, induced apoptosis and caused cell cycle arrest in neuroblastoma cell lines *in vitro*, and reduced tumor growth and extended survival of mice bearing xenografts of cell lines derived from high-risk neuroblastomas. Our data suggest that GSK461364 has the potential to generate a measurable response in patients with high-risk neuroblastomas and might even be considered for rational combination treatment approaches [31]. GSK461364 should, therefore, be considered for entry into early phase clinical trials in pediatric patients.

## MATERIALS AND METHODS

### Compound

GSK461364, (R)-5-[6-[(4-methyl-1-piperazinyl)methyl]-1H-benzimidazol-1-yl]-3-[(1r)-1-[2-(trifluoromethyl)phenyl]ethoxy]-2-thioph-enecarboxamide (Axon Medchem BV, Groningen, The Netherlands) was dissolved in DMSO for a 10 mmol/l stock solution for *in vitro* studies, and stored at -80°C.

### Cell culture

The SK-N-AS, SH-SY5Y, SH-EP, IMR32, Kelly and SK-N-BE human neuroblastoma cell lines were cultivated in RPMI-1640 medium (Invitrogen, Karlsruhe, Germany) supplemented with 10% fetal calf serum (Invitrogen), 100 U/ml penicillin/streptomycin (Invitrogen) and 2.5 mg/l amphotericin B (PAA Laboratories GmbH, Egelsbach, Germany) and were incubated in a humidified 5% CO2 atmosphere at 37°C. Cell line identity was verified by the German Collection of Microorganisms and Cell Cultures (DSMZ, Braunschweig, Germany).

### Cell assays

Neuroblastoma cell lines were seeded onto 96-well plates (2 × 10^3^ cells per well) in triplicate for all assays, and incubated for 24 hours to permit surface adherence. Viability was assessed using the 3-(4,5-dimethylthiazol-2-yl)-2,5-diphenyltetrazolium bromide (MTT) assay (Roche, Basel, Switzerland). Apoptosis and proliferation were assessed using the Cell Death and BrdU ELISAs (Roche), respectively. All assays were performed according to the manufacturer's protocols. For cell cycle analysis, SK-N-AS and IMR32 cells were suspended by trypsinization and washed 3 times with PBS, then incubated with propidium iodide for 15 min to stain DNA. Cellular DNA content was analyzed using a FC500 flow cytometer (Beckman Coulter, Krefeld, Germany). Colony forming ability was assessed in SK-N-AS or IMR32 cells by staining colonies with 1,9-dimethyl-methylene blue (Sigma Aldrich, St. Louis, MO, USA) for 45 minutes, washed twice with PBS and colonies were counted. All experiments were independently performed in triplicates at least 3 times, if not otherwise indicated.

### Gene expression analyses

SK-N-AS cells were seeded onto 6-well plates (1 × 10^5^ cells/well), allowed 12 hours to adhere then incubated 24 hours with 500 nM GSK461364 or control medium (containing 0.1% DMSO). Total RNA was extracted using the RNeasyMini kit (Qiagen, Hilden, Germany), and samples were profiled on the Affymetrix Human Gene Expression Array (HG-U133 Plus 2.0, Affymetrix, Santa Clara, CA, USA), both according to manufacturers’ protocols. CEL files were normalized and summarized to gene levels to conduct gcRMA normalization from the Bioconductor repository of statistical tools [64]. Only probes with the highest mean expression for each gene and with a log2 expression >5 were included in the analysis for differential expression using the Rank Product analysis package v2.36.0 in R [65]. Samples were clustered using hierarchical clustering, and dissimilarity calculated using Manhattan distances. Gene set enrichment analysis was performed using GSEA v2.0 software ( www.broadinstitute.org/gsea) [66]. Genes were ranked by calculating the difference in population means scaled by the standard deviation to generate a signal to noise ranking metric. We used publicly available curated gene sets (c2.all.v4.0.symbols.gmt), gene motif sets (c3.all.v4.0.symbols.gmt) and oncogenic signatures (c6.all.v4.0.symbols.gmt) in our analyses. Newly generated microarray data were deposited in the GEO database (accession no. GSE67102).

### Xenograft model

SK-N-AS and IMR32 neuroblastoma cells were cultured to 80% confluency, harvested and suspended in 200 μL Matrigel™ (BD Bioscience, Heidelberg, Germany) for subcutaneous inoculation of 2 × 10^7^ cells into the left flank of 8-week-old female athymic *nu/nu* mice (n = 12 mice). Mice were randomly assigned (6 mice per group) to either GSK461364 or vehicle (0.1% DMSO, 5% glucose) control groups after tumors reached 200 mm^3^. Vehicle control or 50 mg GSK461364 per kg body weight was intraperitoneally injected every second day until all control mice reached the endpoint (tumor volume of 2,500mm^3^) but not longer than 40 days. The same dose and treatment scheme was chosen as used in previous preclinical GSK461364 studies in various xenograft tumor models, and which was shown to be safe and effective [35]. Tumor growth was monitored using a caliper, and tumor volume was calculated using the formula, (breadth × length × height)/2. Mice were euthanized by cervical dislocation when tumor size exceeded 2,500mm^3^. In mice, whose xenograft tumors were to be immunohistologically examined for apoptosis and proliferation, 2 doses of 100 mg GSK461364 per kg body weight were administered every 12 hours over a 3-day course (n = 3 mice in both GSK461364 and vehicle control groups). Mice were euthanized 4 hours after the last treatment injection, and xenograft tumors were excised, formalin-fixed and paraffin-embedded. All animal experiments were performed in accordance with the Council of Europe guidelines for accommodation and care of laboratory animals, and protocols were approved by the Ethical Commission for Animal Experimentation at the University Hospital Essen.

### Immunohistochemistry

Formalin-fixed, paraffin-embedded tissue sections (5 μm) were deparaffinized using routine techniques, and placed in 200 ml of EnVision™ target retrieval solution (pH 6.0; Dako, Hamburg, Germany) for 20 minutes at 100°C. After cooling for 20 minutes, slides were quenched with 3% H_2_O_2_ for 5 minutes before incubating with primary antibodies against cleaved caspase 3 (#cat 9661; 1:200; Cell Signaling, Danvers, MA, USA) to detect apoptotic cells or marker of proliferation Ki-67 (#cat M724029-2, 1:25, Dako) using a Dako Autostainer (Dakocytomation). Immunostaining was visualized using the EnVision™+ kit (Dako).

### Statistics

Pairs of interval variables were compared with Student's two-sided t-tests using SPSS Statistics for Windows, v21.0. (IBM, Armonk, NY, USA). Error bars in figures represent standard deviation. Graph Pad Prism 5.0 (GraphPad Software Inc., San Diego, CA, USA) was used to calculate GI50 or 80 concentrations and to perform Kaplan-Meier analyses.

## SUPPLEMENTARY MATERIALS FIGURES AND TABLES






