# Supplementary material for: The GSK461364 PLK1 inhibitor exhibits strong antitumoral activity in preclinical neuroblastoma models
Source: Oncotarget. 2016 Dec 27;8(4):6730–41. doi: 10.18632/oncotarget.14268 (PMC5351666; doi:10.18632/oncotarget.14268)
Supplement: Supplementary file 1 [file oncotarget-08-6730-s001.pdf]

Induction chemotherapy for the treatment of non-endemic locally advanced nasopharyngeal carcinoma

SUPPLEMENTARY TABLES

Supplementary Table S1: Univariate analyses of various risk factors for prognosis in 233 patients.

See Suppelemntary File 1

Supplementary Table S2: Toxicities related to different induction chemotherapy regimes.

See Supplementary File 2
