# Supplementary material for: The GSK461364 PLK1 inhibitor exhibits strong antitumoral activity in preclinical neuroblastoma models
Source: Oncotarget. 2016 Dec 27;8(4):6730–41. doi: 10.18632/oncotarget.14268 (PMC5351666; doi:10.18632/oncotarget.14268)
Supplement: Supplementary file 2 [file oncotarget-08-6730-s002.doc]

**Supplemental table 1 Univariate analyses of various risk factors for prognosis in 233 patients**

|  | **NO.** | **5y**  **OS** | **Hazard ratio**  **( 95%CI)** | **P** | **5y**  **DFS** | **Hazard ratio**  **( 95%CI)** | **P** | **5y**  **DMFS** | **Hazard ratio**  **( 95%CI)** | **P** | **5y**  **LFS** | **Hazard ratio**  **( 95%CI)** | **P** |
| --- | --- | --- | --- | --- | --- | --- | --- | --- | --- | --- | --- | --- | --- |
| **Age (yr)** | | | | | | | | | | | | | |
| **＜50y** | 144 | 89.2 | 1 |  | 83.1 | 1 |  | 84.8 | 1 |  | 97.6 | 1 |  |
| **≥50y** | 89 | 82.5 | 2.245(1.130-4.461) | **0.018^*^** | 67.4 | 1.350(0.821-2.222) | **0.237** | 80.7 | 0.784(0.400-1.539) | **0.480** | 90.5 | 2.515(0.668-9.475) | **0.159** |
| **Gender** | | | | | | | | | | | | | |
| **Male** | 171 | 88.9 | 1 |  | 80.2 | 1 |  | 86.5 | 1 |  | 97.2 | 1 |  |
| **Female** | 62 | 87.2 | 0.707(0.306-1.629) | **0.415** | 79.2 | 0.450(0.229-0.884) | **0.020^*^** | 84.9 | 0.510(0.226-1.150) | **0.105** | 98.3 | 0.318(0.040-2.543) | **0.280** |
| **AJCC** | | | | | | | | | | | | | |
| **T1-3** | 140 | 87.9 | 1 |  | 81.1 | 1 |  | 83.6 | 1 |  | 95.6 | 1 |  |
| **T4** | 93 | 86.4 | 1.018(0.505-2.051) | **0.961** | 72.7 | 0.818(0.487-1.374) | **0.447** | 79.6 | 0.901(0.476-1.706) | **0.750** | 98.7 | 0.209(0.026-1.679) | **0.141** |
| **AJCC** | | | | | | | | | | | | | |
| **N0-2** | 178 | 90.8 | 1 |  | 83.2 | 1 |  | 88.6 | 1 |  | 97.5 | 1 |  |
| **N3** | 55 | 79.9 | 1.033(0.462-2.309) | **0.937** | 47.4 | 1.233(0.708-2.149) | **0.459** | 73.9 | 1.075(0.526-2.196) | **0.843** | 78.8 | 1.698(0.424-6.801) | **0.455** |
| **Clinical stage** | | | | | | | | | | | | | |
| **Ⅲ** | 94 | 88.2 | 1 |  | 82.7 | 1 |  | 87.8 | 1 |  | 98.1 | 1 |  |
| **Ⅳa-b** | 139 | 81.3 | 0.865(0.431-1.736) | **0.683** | 67.1 | 0.888(0.542-1.454) | **0.637** | 75.4 | 0.758(0.411-1.400) | **0.376** | 93.4 | 0.642(0.172-2.399) | **0.510** |
| **Stage** | | | | | | | | | | | | | |
| **T1-2N2-3** | 82 | 81.6 | 1 |  | 70.5 | 1 | **1** | 76.1 | 1 | **1** | 98.5 | 1 | **1** |
| **T3-4N0-1** | 31 | 86.2 | 0.646(0.181-2.297) | **0.499** | 78.7 | 0.612(0.252-1.486) | **0.278** | 81.5 | 0.652(0.218-1.954) | **0.445** | 96.3 | 0.405(0.049-3.313) | **0.399** |
| **T3-4N2-3** | 120 | 84.7 | 1.152(0.553-2.401) | **0.705** | 62.6 | 0.868(0.516-1.461) | **0.594** | 75.2 | 1.043(0.541-2.013) | **0.889** | 90.3 | 0.112(0.014-0.913) | **0.041^*^** |
| **Pathological type** | | | | | | | | | | | | | |
| **WHOⅡ** | 61 | 91.7 | 1 |  | 76.5 | 1 |  | 79.0 | 1 |  | 97.5 | 1 |  |
| **WHOⅢ** | 158 | 84.0 | 1.466(0.497-4.325) | **0.488** | 82.1 | 1.515(0.739-3.108) | **0.253** | 88.4 | 1.076(0.491-2.359) | **0.854** | 97.9 | 2.060(0.251-16.892) | **0.510** |
| **Induction chemotherapy regimens** | | | | | | | | | | | | | |
| **PF** | 26 | 63.9 | 1 | **1** | 62.9 | 1 | **1** | 65.3 | 1 | **1** | 88.6 | 1 | **1** |
| **TP** | 129 | 82.7 | 0.512(0.223-1.172) | **0.113** | 62.3 | 0.576(0.300-1.106) | **0.097** | 70.2 | 0.597(0.252-1.414) | **0.241** | 97.4 | 0.644(0.107-3.884) | **0.631** |
| **GP** | 59 | 92.4 | 0.181(0.048-0.675) | **0.011^*^** | 72.9 | 0.368(0.160-0.884) | **0.018^*^** | 77.7 | 0.589(0.219-1.583) | **0.294** | 98.2 | 0.343(0.028-4.222) | **0.404** |
| **Retropharyngeal lymph node** | | | | | | | | | | | | | |
| **No** | 107 | 91.4 | 1 | **1** | 73.5 | 1 |  | 82.2 | 1 |  | 97.4 | 1 |  |
| **Yes** | 126 | 85.3 | 3.513(1.565-7.885) | **0.002^*^** | 77.4 | 1.810(1.085-3.021) | **0.023^*^** | 79.4 | 1.462(0.781-2.735) | **0.235** | 95.1 | 1.448(0.381-5.500) | **0.587** |
| **Invasion of the skull base** | | | | | | | | | | | | | |
| **No** | 164 | 90.3 | 1 | **1** | 82.3 | 1 |  | 86.0 | 1 |  | 97.4 | 1 |  |
| **Yes** | 69 | 77.2 | 3.413(1.692-6.885) | **0.001^*^** | 72.5 | 1.268(0.752-2.138) | **0.374** | 82.4 | 1.050(0.532-2.073) | **0.889** | 95.4 | 0.316(0.039-2.549) | **0.254** |
| **Neck lymph node metastasis** | | | | | | | | | | | | | |
| **None&Unilateral** | 44 | 89.0 | 1 | **1** | 83.7 | 1 |  | 88.3 | 1 |  | 85.7 | 1 |  |
| [**Bilateral**](javascript:void(0);) | 189 | 79.5 | 10.399(1.409-76.765) | **0.022^*^** | 71.8 | 3.171(1.364-7.371) | **0.007^*^** | 74.5 | 1.887(0.791-4.504) | **0.146** | 97.5 | 2.455(0.306-19.715) | **0.769** |
| **Lymph nodes were performed before treatment** | | | | | | | | | | | | | |
| **No** | 213 | 90.7 | 1 | **1** | 85.1 | 1 |  | 89.7 | 1 |  | 97.3 | 1 |  |
| **Yes** | 20 | 81.4 | 0.980(0.233-4.134) | **0.978** | 60.6 | 1.120(0.447-2.805) | **0.808** | 77.7 | 1.116(0.341-3.653) | **0.856** | 75.0 | 1.897(0.231-15.544) | **0.544** |
