# Supplementary material for: The GSK461364 PLK1 inhibitor exhibits strong antitumoral activity in preclinical neuroblastoma models
Source: Oncotarget. 2016 Dec 27;8(4):6730–41. doi: 10.18632/oncotarget.14268 (PMC5351666; doi:10.18632/oncotarget.14268)
Supplement: Supplementary file 3 [file oncotarget-08-6730-s003.doc]

**Supplemental table 2 Toxicities related to different induction chemotherapy regimes**

|  | **PF (No./%)** | | | | | **TP (No./%)** | | | | | **GP (No./%)** | | | | |
| --- | --- | --- | --- | --- | --- | --- | --- | --- | --- | --- | --- | --- | --- | --- | --- |
|  | **0** | **1** | **2** | **3** | **4** | **0** | **1** | **2** | **3** | **4** | **0** | **1** | **2** | **3** | **4** |
| **Anemia** | 26(100.0) | 0(0) | 0(0) | 0(0) | 0(0) | 120(93.0) | 7(5.4) | 2(1.6) | 0(0) | 0(0) | 51(86.4) | 8(13.6) | 0(0) | 0(0) | 0(0) |
| **Thrombocytopenia** | 26(100.0) | 0(0) | 0(0) | 0(0) | 0(0) | 111(86.0) | 7(5.4) | 11(8.5) | 0(0) | 0(0) | 53(89.8) | 5(8.5) | 1(1.7) | 0(0) | 0(0) |
| **Neutropenia** | 1(3.8) | 12(33.3) | 8(30.8) | 5(19.2) | 0(0) | 69(53.5) | 32(24.8) | 26(20.2) | 2(1.6) | 0(0) | 25(42.4) | 15(25.4) | 16(27.1) | 3(5.1) | 0(0) |
| **Febrile neutropenia** | 20(76.9) | 6(23.1) | 0(0) | 0(0) | 0(0) | 124(96.1) | 5(3.9) | 0(0) | 0(0) | 0(0) | 55(93.2) | 3(5.1) | 1(1.7) | 0(0) | 0(0) |
| **Vomiting** | 11(42.3) | 15(57.7) | 0(0) | 0(0) | 0(0) | 35(27.1) | 78(60.5) | 16(12.4) | 0(0) | 0(0) | 21(35.6) | 33(55.9) | 5(8.5) | 0(0) | 0(0) |
| **Hand–foot syndrome** | 0(0) | 0(0) | 0(0) | 0(0) | 0(0) | 0(0) | 0(0) | 0(0) | 0(0) | 0(0) | 0(0) | 0(0) | 0(0) | 0(0) | 0(0) |
| **Ototoxicity** | 21(80.8) | 5(19.2) | 0(0) | 0(0) | 0(0) | 114(88.4) | 15(11.6) | 0(0) | 0(0) | 0(0) | 48(81.4) | 11(18.6) | 0(0) | 0(0) | 0(0) |
| **Neuropathy** | 0(0) | 0(0) | 0(0) | 0(0) | 0(0) | 0(0) | 0(0) | 0(0) | 0(0) | 0(0) | 0(0) | 0(0) | 0(0) | 0(0) | 0(0) |
